# Examination of the usability of Tinkercad application in educational robotics teaching by eye tracking technique

**DOI:** 10.1186/s40561-023-00242-0

**Published:** 2023-03-23

**Authors:** Rumeysa Erdogan, Zeynep Saglam, Gulay Cetintav, Fatma Gizem Karaoglan Yilmaz

**Affiliations:** 1 Department of Computer Technology and Information Systems, Faculty of Science, Bartin University, Bartın, Turkey; 2 Graduate School, Bartin University, Bartin, Turkey

**Keywords:** Arduino, Tinkercad, Mikro:bit, Usability, Robotic-coding

## Abstract

Like all sectors, the education sector has been negatively affected by the Covid-19 pandemic. Considering the decision to conduct face-to-face training in schools remotely, teachers had many difficulties in moving course content to the online platform. Teachers who perform robotic coding applications are looking for ways to do these activities remotely. Simulators enable real objects to be animated in a computer environment. There are many paid and free platforms that simulate robotic coding tools. Arduino and Micro:bit, can be simulated on the Tinkercad platform. This study tests the usability of the Tinkercad platform by teachers. In usability tests, users are expected to complete the authentic tasks they must perform on the tested platform. In this study, 12 Information Technology teachers tried to complete 10 authentic tasks in the Circuits section of the Tinkercad platform. Eye movements have been recorded and analyzed while participants perform tasks. Surveys were applied to the participants and data were collected with the observation form during the tests. Consequently, the teachers who completed the usability tests stated that it is appropriate for the platform to be used by teachers and students over 10 years of age and that they can use the platform in their activities.

## Introduction

### Coding and the world of the future

Countries are investing in many areas to develop, to have a say in the world. All investments and expenditures are to become an improved and self-sufficient country. The development of a society can only be with qualified human power (Sayin & Seferoglu, 2016). In the current century, the concept of qualified human power changes definition every day, and the concept of 21st- skills is a concept related to qualified human power. In the increasingly digitalized world, it is very important that children are equipped with 21st- skills (Baz, 2018). Problem-solving, creativity, innovation skills, keeping up with new situations, working with a group, critical thinking, and technology literacy are the competencies to be achieved today (Partnership for 21st Century Learning, 2019; Yilmaz et al., 2020). People who have improved themselves and who have the potential and ability to be a producer rather than a consumer in a technological sense are qualified people. Countries will develop more technologically when the number of these people is high.

While it is important to innovate the education system and content for children to have these skills, it is equally important that teachers develop themselves in this area. Ministry of National Education (MEB) (2018) announced in its 2023 vision document that students, teachers, and administrators at all levels of education will be given production skills with informatics such as 3D design, coding, and electronic design. The background commands within the technological devices we use, which enable programs to work, are created through coding (Sanal & Erdem, 2017). In this context, coding education in schools is very important, developed countries are making the necessary importance and investments (Sayin & Seferoglu, 2016).

Concepts such as artificial intelligence, the internet of things, cybersecurity, intelligent systems and cities, autonomous systems, and dark factories brought with them the concept of digital transformation (Firat & Firat, 2017). It is said that digital transformation changes professions, while eliminating some existing professions, it will create new professions that do not exist today (Cark, 2020). It is very important that children are ready for the life of the future and especially manage these systems, one of the most important objectives of education is to prepare them for the business world (Yalcin, 2018). Technology education for children is very important in this context. With coding training, children will be aware of the innovations of the industry 4.0 concept and take steps to learn how to manage new technologies.

The concept of coding brings with it the concept of robotics and its education. Especially when children see the results of the codes they write in a concrete way, they are more interested and curious about the subject and learn better through fun activities. Since programming is an abstract concept, it is especially difficult for young children to learn, but embodying these concepts with robotic tools will make learning easier (Ersoy et al., 2011). In their study, Butuner and Dundar (2018) investigated the usability of robotic tools by teachers and examined the most used tools such as Arduino, Micro:bit, O-bot, mBot, and found that the tools were useful. There are different tools for children of all ages to learn robotics and coding. While coding used to be necessary to find scenarios, it is more important to choose the right tool today (Prensky, 2005). While it used to be necessary to find scenarios for coding, today it has become more important to choose the right tool (Prensky, 2005). Many factors are considered by teachers in the selection of tools, the cost of the tool, the ability to reuse, the availability of its side equipment, and easy to code are some of these factors. Identifying and using tools that will contribute to the development of the child can be carried out by experienced and interested teachers.

### Microcontroller cards

#### Micro:bit

**Fig. 1:**
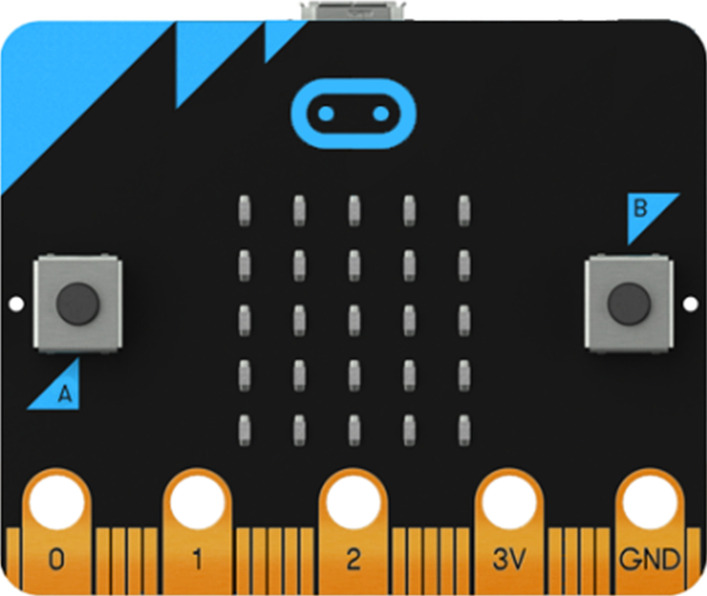
Micro:bit microcontroller card

Micro:bit is a microcontroller card developed with the support of the BBC. The card has a 5 × 5 LED matrix, Bluetooth antenna, 2 buttons, digital and analog input pins, acceleration tilt and compass sensors integrated. It can be programmed with JavaScript and Python, or it is possible to program it on online platforms. Teachers were 85% satisfied with the use of cards given to 11- and 12-year-olds in England in 2016 and found it amusing for students (Vostinar & Kneznik, 2020). Figure 1 also includes an image of a Micro:bit.

The Micro:Bit card is a very useful coding card for younger students thanks to its built-in sensors. Teachers can easily use these cards for students who want to learn robotic coding, who are confused about electronics, and who have difficulty in making connections.

#### Arduino

**Fig. 2:**
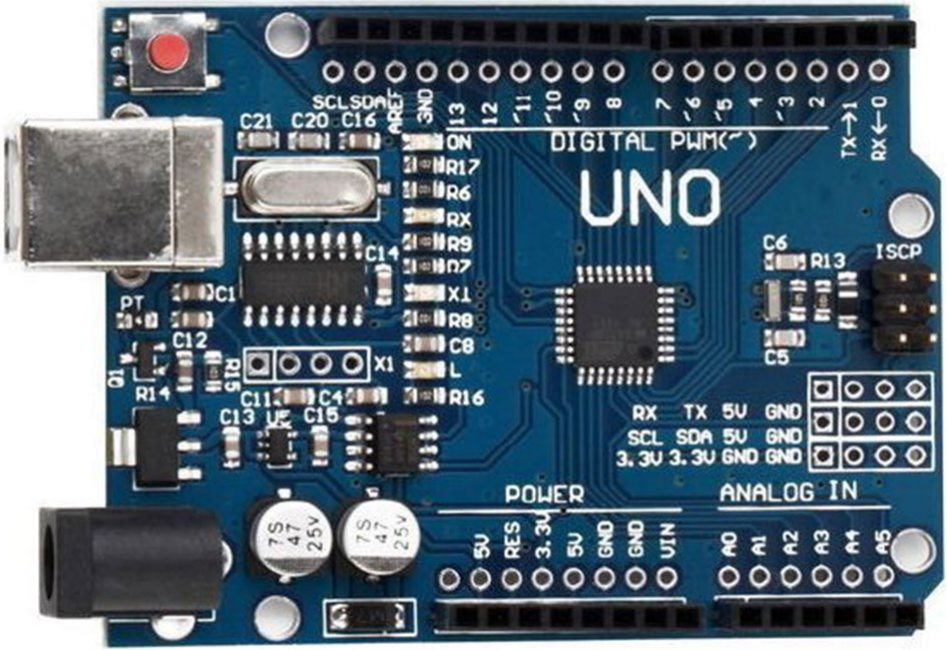
Arduino Uno microcontroller card

Arduino is an open-source microcontroller card. It has digital and analog input and output pins on it. Values can be taken or sent from sensors through pins. It is possible to carry out many intelligent systems, robotic studies, and electronic projects by loading the generated codes onto the card. Arduino can be used at different age levels because it can be encoded with block-based and text-based coding tools. It is often preferred in schools as it is easy to program and has a low cost. There is an image of Arduino Uno in Fig. 2.

Arduino is a microcontroller with many models. The most common model is the uno model. Many projects can be done with this model. There are no built-in sensors on the Arduino. It is important to have basic electronics knowledge to be able to use it, it can be difficult for children to learn in very young age groups.

For beginners in robotic coding, it may be more effective to start with micro:bit and continue with arduino. Robotic coding learning is a process that needs to be learned practically with hardware. However, it can be difficult to reach the equipment due to financial difficulties. There are simulation tools for teachers who want to teach robotic coding but do not have the equipment. By making use of these simulation tools, basic activities can be performed.

### Robotics-coding simulators and tinkercad

Certain hardware is required to study robotic coding. However, it is not possible for individuals who do not have robotic tools to be deprived of this training, circuit design and coding and operation of the designed circuit can be done on simulators. Simulators are tools that allow learning to be done repeatedly in a safe environment, developed to save time, resources, manpower, and life (Saral & Topcu, 2008). Simulators are especially used in dangerous work, and highly cost robotic designs (Cavas & Agrali, 2020). Today, when technology is renewed day by day, it has become very important to use simulators for computer technology training, this situation is inevitable because the life of a technology taken as hardware is very short (Wolffe et al., 2002). Nowadays, there are many free and paid simulators developed for use in training. Educators research and try to use the most suitable tool for themselves and their students.

Tinkercad is a tool originally developed by Autodesk as an online platform for young children to produce 3D materials, teachers, and students who make designs to make and remove simple tools today when 3D printer technology is widespread. The circuits section added to the platform is installing and operating robotic circuits. Circuits installed with Arduino and Micro:bit microcontrollers and sensors can be encoded and simulated as blocks or text depending on the preference. There are sufficient hardware plug-ins to provide basic robotic coding training. The Tinkercad platform is a free-to-use cloud-based system that can be accessed from anywhere. Students and teachers can continue to connect and edit their circuits and designs wherever they want, record their work, and share them if they want. There are course contents, materials shared by users, and sample projects that students and teachers can benefit from on the platform. Teachers can become members of the platform, create their own classes, and include their students in their own classrooms without being a member of the platform with the code they will give. This creates an advantage for students who do not have an e-mail address. Circuits are taken to the installation area by drag-and-drop method, connections can be made using the mouse.

### Usability and usability in robotic coding

With the rapid development of technology, its importance in our lives has increased. Nowadays, the increasing intensity of work, the fact that people aim to save time, the economy, and want to carry out their work independently of space make the importance of technology more and more important every day. Especially with the Covid-19 pandemic, many workplaces and government agencies have switched to flexible working, and staff has started to carry out their work remotely. Most educational institutions have decided to study remotely. As a result of these decisions and practices taken due to the pandemic, a much larger number of people were obliged to use technology. With the frequent use of technology in all areas Human–Computer Interaction (HCI) workspace and usability assessments are needed more. Usability is the ability to easily use a system or equipment, as well as the ability to perform the tasks requested by trained and supported people (Shackel, 2009).

Human–Computer Interaction (HCI) is defined as an interdisciplinary field of study that deals with the design, evaluation, and implementation of technologies (Acarturk & Cagiltay, 2006). It is possible to encounter many definitions of usability. Usability is the general name of a number of methods based on the examination of the interfaces of designs (Nielsen, 1994). Evaluators who want to identify problems in product designs and develop solutions to these problems are doing usability studies. Usability can be measured by a combination of efficiency, effectiveness, and satisfaction shown by users in performing tasks (Acarturk & Cagiltay, 2006).

It is not easy for users to choose a platform that they can use among many options. Teachers prefer useful platforms that have been tried by teachers before. Learning to use a platform requires a certain amount of time, the fact that the learned platform is useless for teachers and students leads to wasted time to learn that platform. Usability studies guide teachers in this sense. During the research, the most used features of the platform are tested by users. As a result of these tests, it is possible to make a judgment about the usability of the platform. Usability research will enable teachers to allocate their time in the process of platform research so that they can use it more efficiently. When the literature was examined, the usability studies of many online platforms were seen. However, the usability of robotic coding tools is not very common.

In their study, Lima Sobreira et al. (2020), explored the usability of block-based coding tools in the Internet of Things (IoT) contexts for the first engineering courses. The Snap4Arduino (S4A) and the App Inventor are discussed in scratch-based applications from block-based encoding tools. The study included five participants to test the interface usability of the programs. Three tasks for S4A and three tasks for the App Inventor have been assigned and participants were asked to perform these tasks. All participants completed these tasks under the same conditions and in the same environment. After the application, an action evaluation survey and software evaluation survey was applied to the participants. When the results were analyzed, it was observed that snap4Arduino and App Inventor applications were usable to engineering students.

In the research conducted by Karaoglan Yilmaz et al. (2019), the information search processes of students were examined through eye monitoring in the Educational Information Network. The data of the study, which was participated by 10 students at the secondary school level, were obtained using surveys and eye tracking reports. It has been observed that students have successfully completed 11 tasks that are thought to be frequently used in the Educational Information Network and have reached the search results correctly and quickly.

In their studies, Pala et al. (2017) aimed to determine how effective the content of the Educational Information Network site is, how satisfied users are and what internal problems are. The participants consist of seven teachers from different branches. The participants were interviewed in advance and then asked to perform eight authentic tasks that were thought to be used most frequently on the Learning Computing Network. An eye tracking device was used in the application Based on the data, it was concluded that there were the most problems in the videos section of the Education Information Network. and the voices of the participants were recorded. In addition, it was emphasized that the course contents were not sufficient in the interviews with the participants.

In their research, Kuzgun and Ozdinc (2017) aimed to measure the usability of Edmodo, the social learning platform, and to uncover existing problems and develop solutions for these problems. In their studies, they included six undergraduate students in Computer and Instructional Technology education department, taking into account the criteria of Edmodo experience. Ten-point authentic tasks were determined and participants were asked to perform these tasks. Participants were observed performing tasks, difficulty, asking for help, or simple tasks were noted. In addition, the "System Usability Scale (SUS)" developed by Brooke (1996) and translated into Turkish by Cagiltay (2011) was also used in the study. SUS and observation results have created the data for the research. When the SUS results were analyzed, Edmodo's usability was determined as medium level.

Kirmaci and Izmirli (2017) have conducted a study to measure the usability of the Online Tracking and Evaluation System (OTES) of Book Reading Activities. Semi-structured interview forms and survey forms were preferred as a data collection tool in the study, which included six class guidance teachers, eight students, and seventeen parents as participants. Researcher logs have been created for the validity and reliability of the research. When the data of the study were examined, it was concluded that the books recommended by OTES were suitable for student levels and interests. Additionally, it has been stated that OTES contributed to the student’s understanding of the books they read.

In the "Public Institution Websites and Usability" study conducted by Durmus et al. (2012), it was aimed to address public institution websites and to identify problems and offer solutions, if any. The websites of 33 public institutions in Turkey have been reviewed. Eight people have been interviewed who are responsible for the design of these sites. A survey of 21 questions was used with the website Assessment Tool, which consists of 18 sections and 102 questions as a data collection tool. As a result of the study, many problems were encountered. Some of these problems include websites are not user-oriented, all kinds of services are not available on the website, the user spending too much time finding the service they need, some sites cannot be searched, or the search function does not give the desired result, some links are dysfunctional or do not work at all. In the study, solutions to the problems of websites are presented. Frequently asked questions on sites, feedback to users, adding online help, search functionality on multiple pages and working are some of the recommended solutions for high-security measures.

In their study, Milosz and Chmielewska (2020) examined the usability of E-Government services using different methods. The group of participants consists of eight people who have never used the system before. In addition to eye tracking, video recording, and voice thinking techniques, it was used in user scenarios and surveys.

### Purpose of the research

In this study, the usability of the Tinkercad platform by Information Technologies (IT) branch teachers is investigated. The teachers who can use the platform most at the point of teaching the use of technology to students are considered as IT Teachers. In the research, teachers are expected to complete authentic tasks and the eye tracking method is used to record the gaze data. Before and after eye tracking, opinions and information of teachers about the platform are taken. Based on the analyzed data, it is aimed to measure the usability of the Tinkercad circuits section in terms of effectiveness, efficiency, and satisfaction. A study that measures the usability of robotic coding tools is not found in the literature; this study will be the first usability study in this scope. The research searches for answers to the following questions:

1. What is the use of Tinkercad platform by Information Technologies Teachers in lessons and online education?
2. What is the status of performing the authentic tasks given in the research?
3. How are the eye movements of the teachers participating in the research while performing their tasks?
4. What is the availability of the platform for teachers and students?
5. At what age level is the Tinkercad program suitable for students in Turkey?

### Importance of research

As a result of the Covid-19 epidemic affecting the whole world, it has been decided to conduct education remotely in Turkey as well as in other countries (Can, 2020; Ministry of National Education, 2020). The course materials used when processing face-to-face courses were inadequate in remote courses and teachers had to edit their contents (Keskin & Ozer, 2020). While teachers teach robot coding with tools in their schools, students who stay away from the tool during the distance education process are not able to understand the activities. Equipment used in robotics activities is costly and the supply of materials is difficult for learners. At this point, robotic simulation tools come into play. Tinkercad is one of the platforms used in this area and can be used for the installation and coding of circuits with Arduino and Micro:bit microcontroller cards. Teachers who teach robotic coding but do not have sufficient tools, or who do not want to break robotic coding training during distance education can work with their students using the circuits section. There are many simulation tools that can be used to teach robotics coding. Tinkercad platform has some features that stand out compared to other platforms. First of all, it does not require installation on a computer, the platform works online; It is suitable for students who attend classes with devices such as phones and tablets without a computer. Teachers can create a classroom on the system by creating a teacher account, and with a code, they can include their students in this class and follow their students' studies in their classrooms. The platform also has a 3D design section. Circuits are easy to install, and one-to-one circuit designs can be made in real life by drag-and-drop. For the programming of the designed circuits, if desired text, and block coding can be used if desired. Although it has these plus features, the usability feature of the platform is a matter of curiosity. It is very important to find a useful platform for robotic coding courses that can be given online, especially during the distance education period. In this research, it is aimed to recommend a tool that teachers can use for this purpose. As a result of the researches, no research was found on the usability of robotic coding simulation tools, and it is thought that the study will fill a gap in this field.

## Method

Within the scope of the study, teachers were asked to open the circuits section on Tinkercad and do pre-determined authentic tasks. During the performing of authentic tasks, the behavior of teachers regarding the application was recorded with the eye tracking device, and the information about the tasks was recorded by observers by filling out observation forms held during the implementation. Users were asked to fill out surveys before and after the application, and their demographic information and opinions about the application were taken. The research is a case study with qualitative and quantitative research techniques.

Robotic coding tools enable students to learn to code while also improving their problem-solving skills. The learning steps of robotic coding lessons, which are increasing in popularity day by day, are generally from simple to complex, from easy to difficult. Robotic coding tools continue with the use of circuits after recognizing the general features of the tool as a first step. It is very important to establish the circuits primarily as hardware and to make the connections correctly. Then, depending on the characteristics of the established circuit, appropriate codes must be written and run. In case the circuit cannot perform the desired task, it is first checked whether the connections are correct and then whether there are any errors in the codes. In this way, students also gain experience in debugging. When going from simple to complex, easy to difficult, the first learning outcome in robotic coding tools is usually LED lighting. Within the scope of this research, usability tests were conducted by using the most frequently shown course activities to the students. When choosing authentic tasks, the activities that the teachers made the students do were selected. The event titles are as follows;

- Led on/off activity
- Traffic light activity
- Potentiometer and led intensity control with Arduino
- LED control with two buttons
- DC motor control

These activities were tested using the most widely used arduino and micro:bit robotic coding tools. The aim here is to test whether these basic activities can be done easily using Tinkercad.

### Process of the research

**Fig. 3:**
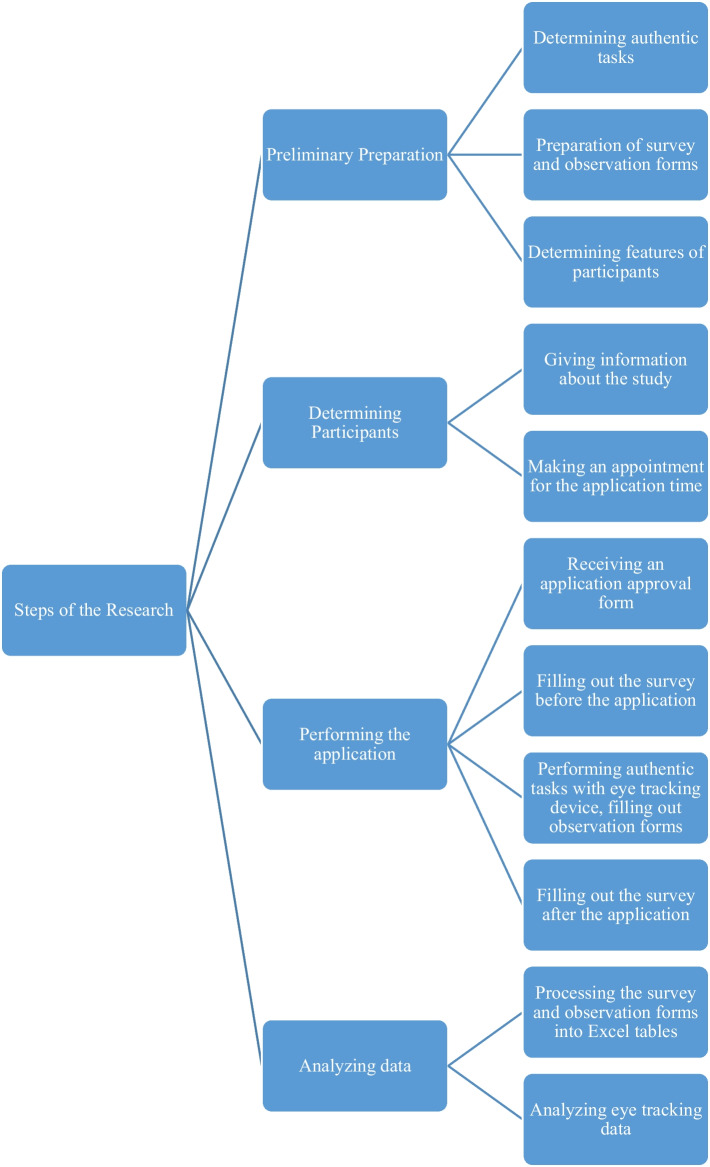
Steps of the research

Figure 3 shows the research process. In order to carry out the research, it was first investigated how to do a usability study and it was concluded that it was appropriate to make users perform authentic tasks. After the authentic tasks were determined, it was ensured that the information that should be collected from the users and important for the research was extracted and pre- and post-application surveys were prepared. Besides, an observation form was prepared to observe the participants during the application. After these preparations, it was researched who would be suitable for the application, and participant characteristics were determined.

After determining the participant characteristics, the participants were contacted and informed about the application and an appointment was given for the application time. On the day of the application, first, a signed consent form was taken from the participants in order to record audio and video and use their information for the study, and they were asked to fill out a pre-application survey that collected preliminary information about the demographic and application. For the application, the participants were calibrated on the computer and asked to perform the tasks in order, while the participants were performing the task, the observers took note of the observation forms and the different situations they encountered during the application. After the application was completed, the participants were asked to fill out a survey after the application, and their opinions and recommendations regarding the application were received.

All the data collected from surveys and observation forms are processed into the excel table, and the appropriate data is analyzed by the table and presented in tables or graphs. The data recorded with the eye tracking device were examined, and as it was not possible to share all the data in the study, interesting ones were shared according to the characteristics of the study.

### Participants

The aim of the research is to test the usability of the Tinkercad circuits department by information technology teachers, accordingly, the participants are IT Teachers. According to the model of Nielsen and Landauer (1993), at least 5 users must participate in the test in usability research. In this study, a total of 12 teachers of 4 women and 8 men in Information Technology volunteered, and it was noted that they had worked with Arduino before. Table 1 demonstrates the demographic information of the participants such as age, gender, and years of seniority. 9 of the teachers are graduates of the Faculty of Computer and Instructional Technologies (FCIT) of universities and 3 are graduates of the Faculty of Technical Education (FTE). 8 teachers work in secondary school, and 4 teachers work in vocational high school / high school. 5 teachers worked for more than 10 years and 7 teachers worked for less than 10 years. 11 of the participants work in Caycuma in Zonguldak and 1 in the Center of Bartin.

**Table 1:** Demographic characteristics of the participants

Participants	Gender	Age	Years of seniority	Graduation faculty	Type of working school
Participant 1	Man	34	8	FCIT	Secondary school
Participant 2	Woman	34	8	FCIT	Secondary school
Participant 3	Woman	41	17	FCIT	High school
Participant 4	Woman	37	15	FCIT	Secondary school
Participant 5	Man	35	6	FCIT	Secondary school
Participant 6	Man	31	7	FCIT	Secondary school
Participant 7	Man	36	16	FCIT	Secondary school
Participant 8	Man	31	7	FCIT	Secondary school
Participant 9	Man	29	5	FCIT	High school
Participant 10	Man	40	18	FTE	Vocational high school
Participant 11	Woman	32	7	FTE	Secondary school
Participant 12	Man	34	12	FTE	Vocational high school

### Data collection tools

#### Survey

Prior to the application, demographic information such as age, gender, graduate department, the school where he/she worked, teacher seniority, as well as information about their use of robotic coding tools was also obtained from the participants. Whether they have used the Tinkercad platform before, their frequency of use has been learned. After the application, the opinions of participants on the tasks given, the difficult situations of the tasks, whether Tinkercad can be used for simulation purposes, and their recommendations to students and teachers were collected.

Research has been conducted on how to collect information from participants before and after the application, similar studies have been reviewed, scales used, and surveys have been analyzed. Authentic tasks were made into a list of important information that should be collected considering the characteristics of the test and insignificant, repetitive information was removed from the list. The prepared list was finalized to the appropriate survey views and opinions were obtained from three field experts and it was concluded that the surveys were appropriate.

#### User test

To examine the usability of the Tinkercad platform, 10 authentic tasks that participants must perform have been determined. When determining tasks, it was noted that they are the most used tasks in robotic coding teaching. It is possible to categorize tasks as very easy, easy, difficult, and very difficult. When determining the degree of ease and difficulty, the complexity of the circuit connections, and the difficulty of coding, are considered. When determining the tasks, the opinions of 3 field experts and 3 people who had previously used Tinkercad were taken, and it was decided that the tasks were appropriate.

The completion times of the participants, and their completion statuses are processed in the observation form, and those who are missing the task are marked as incomplete. The authentic tasks and their difficulty levels are shown in Table 2.

**Table 2:** Authentic tasks and difficulty ratings

No	Difficulty rate	Task
1	Very easy	In the circuits section, set up a "single LED circuit" and run the simulation by writing codes to turn on this LED at intervals of 1 s
2	Easy	In the circuits section, set up a "traffic light circuit" and write the codes and run the simulation so that the red-yellow-green lamps will turn on for one second, then the other lamp will turn on while one is off
3	Difficult	In the Circuits section, set up the "circuit that controls the led intensity with a potentiometer", write the codes and run the simulation
4	Easy	In the circuits section, set up the "DC motor to start" circuit and start the motor clockwise for one second, opposite direction for 1 s (it is okay if the order is reversed), then write the codes that stop it and run the simulation
5	Very difficult	In the Circuits section, set up a "Two buttons and one led" circuit, write the codes that allow the LED to turn on when one button is pressed, and the LED to turn off when the other button is pressed, and run the simulation
6	Very easy	In the Circuits section, write the codes that turn on the 1st led on the Micro:bit card at intervals of 1 s and run the simulation
7	Very difficult	In the circuits section, set up a "traffic light circuit" and write the codes and run the simulation so that the red-yellow-green lamps will turn on for one second, then the other lamp will turn on while one is off
8	Difficult	In the Circuits section, set up the "circuit that controls the led intensity with a potentiometer", write the codes and run the simulation
9	Easy	In the circuits section, set up the "DC motor to start" circuit and start the motor clockwise for one second, opposite direction for 1 s (it is okay if the order is reversed), then write the codes that stop it and run the simulation
10	Very easy	In the Circuits section, write the codes that allow the LED in 1st row to turn on at the top when press the A button on the Micro:bit card, and to turn off when the B button is pressed, and run the simulation

#### Observation form

It is a form where participants are observing them when performing authentic tasks, how much of the tasks they have completed, completion times, and if there is a special case, the explanations are noted. While creating the form, the opinions of field experts about the form were received and the deficiencies were completed.

#### Eye tracking data

The participants were informed before the application about the test to be performed on them and participated in the research voluntarily. Approval has been obtained for the use of the data collected from them. The participants came to the test by appointment and applied one by one. During the application, the eye-tracking data of users were recorded with the eye-tracking device while performing the task. With the eye tracking technique, it can be recorded how much users look at which point and what areas they focus on (Karaoglan Yilmaz & Yilmaz, 2019). 4 different data were obtained from each user, including the eye tracking video, heat map image, eye splash picture, and eye splashes heat map. The device used during eye tracking is a device called Tobii PCEye Mini and the software used is Gaze Viewer. In addition, pre-test calibration was performed for each participant, Windows Control software was used for calibration.

### Analyzing data

Survey data obtained from users before and after application, observation forms held by observers during performing tasks, and data obtained by eye tracking device were evaluated and the results were analyzed. The data recorded by the eye tracking device has been reviewed, comparing the times to perform tasks, making tasks easy and difficult. Other collected data have been processed into a form in excel and tables have been created. The completion times of the tasks, which users complete them, the average time allocated to each task, and the completion rates of the tasks are table and chart. The duration, completion status, total time spent, and average durations of each task for each task are tabled and examined. The SPSS package program was used to analyze whether there is a difference in the time spent by the participants whose seniority is more than 10 years and those with less than 10 years while performing their duties.

## Findings

The completion status and duration of the 12 participants in our research are recorded in seconds and shown in Table 3.

**Table 3:** Duration of participants to complete tasks

Participants	T 1	T 2	T 3	T 4	T 5	T 6	T 7	T 8	T 9	T 10
Participant 1	52	128	143	272	247	32	188	180	97	43
Participant 2	91	159	142	114	–	32	176	124	68	45
Participant 3	77	185	107	71	203	25	141	–	65	44
Participant 4	248	206	115	95	–	72	209	–	94	81
Participant 5	206	304	245	127	307	70	254	195	73	78
Participant 6	575	303	–	–	–	–	403	208	76	60
Participant 7	95	180	171	99	–	40	196	167	88	72
Participant 8	171	286	263	195	306	35	246	249	80	52
Participant 9	71	135	132	74	196	15	159	–	69	61
Participant 10	117	190	–	108	227	22	131	–	61	53
Participant 11	207	230	138	93	–	76	243	105	93	102
Participant 12	138	172	148	114	951	58	185	171	121	71
Average duration of tasks	171	207	160	124	348	43	211	175	82	64

^*^T Task

When Table 3 is examined, it is seen that the most unsuccessful task is Task 5, which is in the "very difficult" category, 5 out of 12 participants could not complete this task, and the reason for not completing the task is usually due to the complexity of the connections and the excess of circuit elements, some of the participants could not remember their codes although they could complete the connections. The second task that could not be done at most was Task 8, which is in the "difficult" category. Participants who can complete the circuit setup of this task often could not complete the task because they could not write their codes. Tasks 1, 2, 7, 9, and 10 were successfully completed by all participants. The application that the participants do in the shortest time on average is Task 6, and the application done in the longest time is Task 5.

**Fig. 4:**
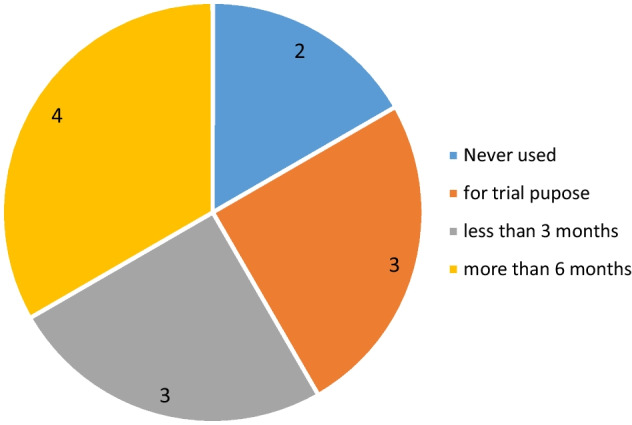
Answers to the question of how much you have used Tinkercad

In the pre-test survey questions, the participants were asked whether they have used the Tinkercad platform before. Figure 4 shows the graphical representation of the answers given to the question.

When Fig. 4 is examined, it is determined that 2 participants have never used the platform before, 4 participants used the platform for more than 6 months, 3 participants for less than 3 months, and 3 participants for trial purposes.

**Fig. 5:**
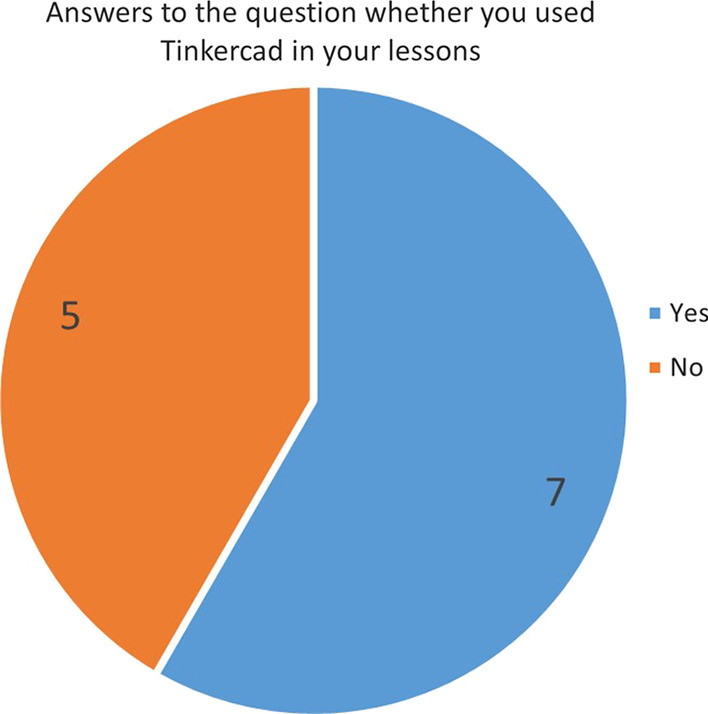
Answers to the question whether you used Tinkercad in your lessons

The other question asked to the participants before the test was whether they have used the Tinkercad platform before in their lessons. Figure 5 shows the number of answers given to this question.

When Fig. 5 is examined, it is seen that 7 participants have used it in their lessons before, and 5 participants have not used it. Of the participants who answered yes, 1 used only the 3D design section, 2 only used the circuits section, and 4 used both the 3D design and circuits section in their lessons.

Table 4 shows how many tasks were successfully completed by the participants, what percentage of the tasks were completed correctly, and the total time in seconds and minutes they spent doing the tasks.

**Table 4:** Status of the participants to complete the tasks

Participants	Number of tasks completed	Percentages of completing tasks (%)	Total time of task completion (seconds)	Total time of task completion (minutes)
Participant 1	10	100	1382	23
Participant 2	9	90	951	16
Participant 3	9	90	918	15
Participant 4	8	80	1120	19
Participant 5	10	100	1859	31
Participant 6	6	60	1625	27
Participant 7	9	90	1108	18
Participant 8	10	100	1883	31
Participant 9	9	90	912	15
Participant 10	8	80	909	15
Participant 11	9	90	1287	21
Participant 12	10	100	2129	35
Averages of participants	9	89	1340	22

When Table 4 is examined, it is seen that at least 8 of the tasks except 1 person were successfully completed, it was learned that these participants took an active role in the Robotics and Coding project carried out by the Zonguldak Governorship 2 years ago and exhibited products in the robot exhibition with their students. Participant 6, who successfully completed 6 tasks, is working in Bartin province, this participant did not do robotic studies with his students, he did robotic studies in line with his own interest, he used the Tinkercad platform more than 6 months ago for trial purposes only. Although the participants have worked with Arduino before, they have never used Micro:bit, but since the use of this board is very simple compared to Arduino, they did not have difficulty in setting up and coding the circuits. Participants successfully completed 9 tasks on average, 89% of the tasks. The participant who spends the most time while performing tasks is Participant 12 with 35 min. Participants 3, 9, and 10 spent the least amount of time by performing the tasks in 15 min, but these participants completed 9 tasks. The average time the participants spend completing the tasks is 22 min.

**Fig. 6:**
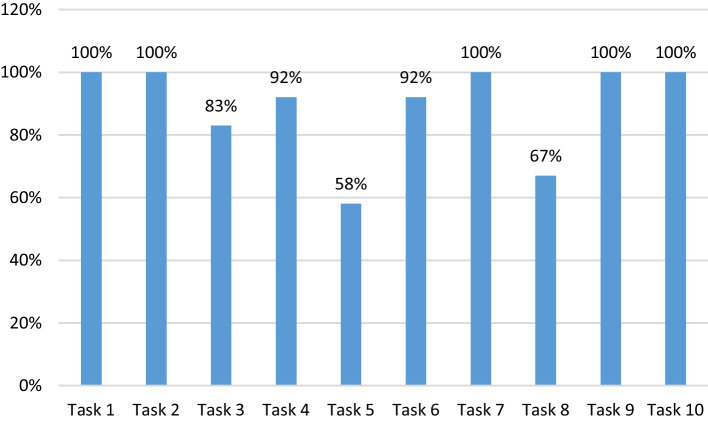
Rates of task completion

The graph in Fig. 6 shows the completion rate of each task.

When Fig. 6 is examined, it is seen that the 5 tasks are 100% completed, and the completion rate of Task 5, which is one of the two tasks in the "very difficult" category, remains at 58%. It is seen that Task 7, which is in the "very difficult" category, has been successfully completed by all participants. The second least completed task is Task 8 with a 67% completion rate.

The seniority year of 5 of the 12 participants participating in the study is over 10 years, and the others are under 10 years. In particular, in terms of using new technologies, it is wondered whether there is a difference between the teachers who started teaching later and those who did this job for more than 10 years. For this purpose, the completion times of tasks are discussed in order to make comparisons on the data. It was tested whether there is a difference in the time to complete tasks between teachers newer than 10 years and those with seniority of more than 10 years. Due to the small number of participants, the Mann–Whitney U test was used to compare the two groups. As a result of the analysis, it is observed that there is no significant difference between the teachers with seniority years over 10 years and teachers less than 10 years in terms of the time to complete the tasks (*p* = 0.432).

**Fig. 7:**
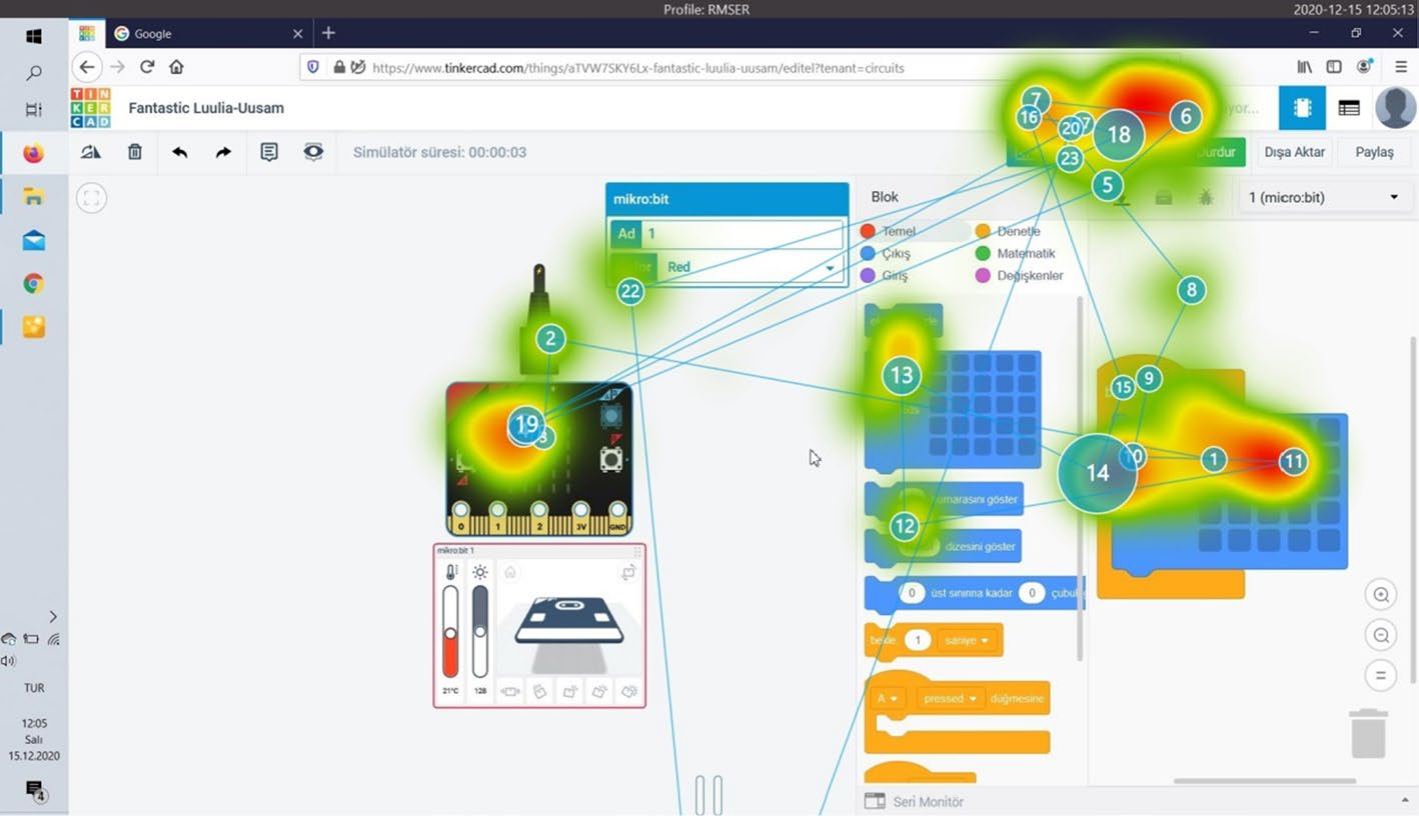
Task 6 heat and splash map of participant 9

Eye tracking data provides information about where the users look on the screen while performing their tasks, and the order in which they perform the operations in the tasks they will perform. Among the authentic tasks assigned to the users, the task completed in the shortest time on average and by 9 participants is Task 6. The heat and splash map of Participant 9, which does not require circuit setup, but performs this task performed only with codes, in a short period of 15 s, is given in Fig. 7. There is very little eye splash in the image. The participant focused on the codes and the simulation button and then the LEDs on the Micro:bit card.

**Fig. 8:**
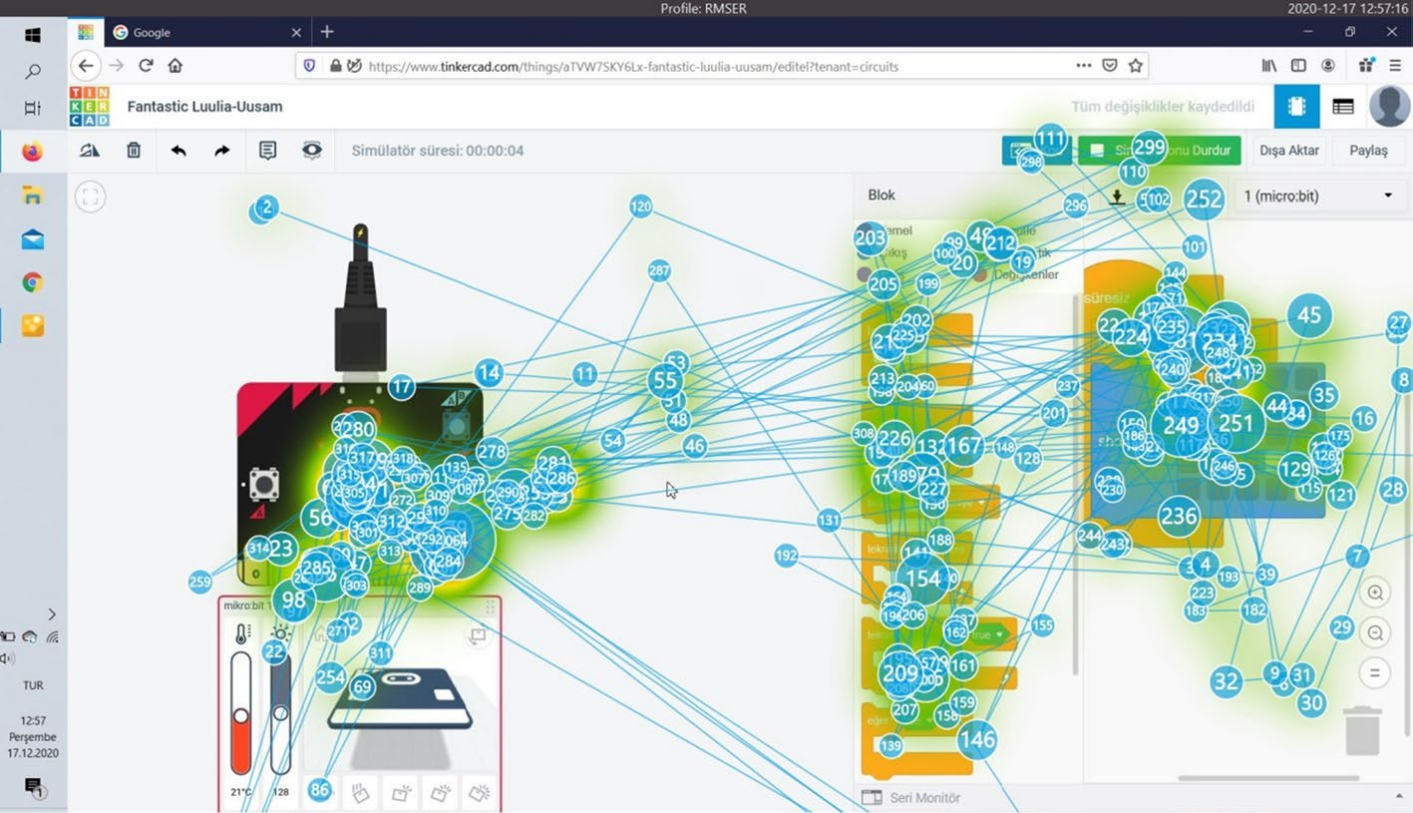
Task 6 Heat and splash map of participant 6

Figure 8 is for Participant 6 who could not complete the same task.

**Fig. 9:**
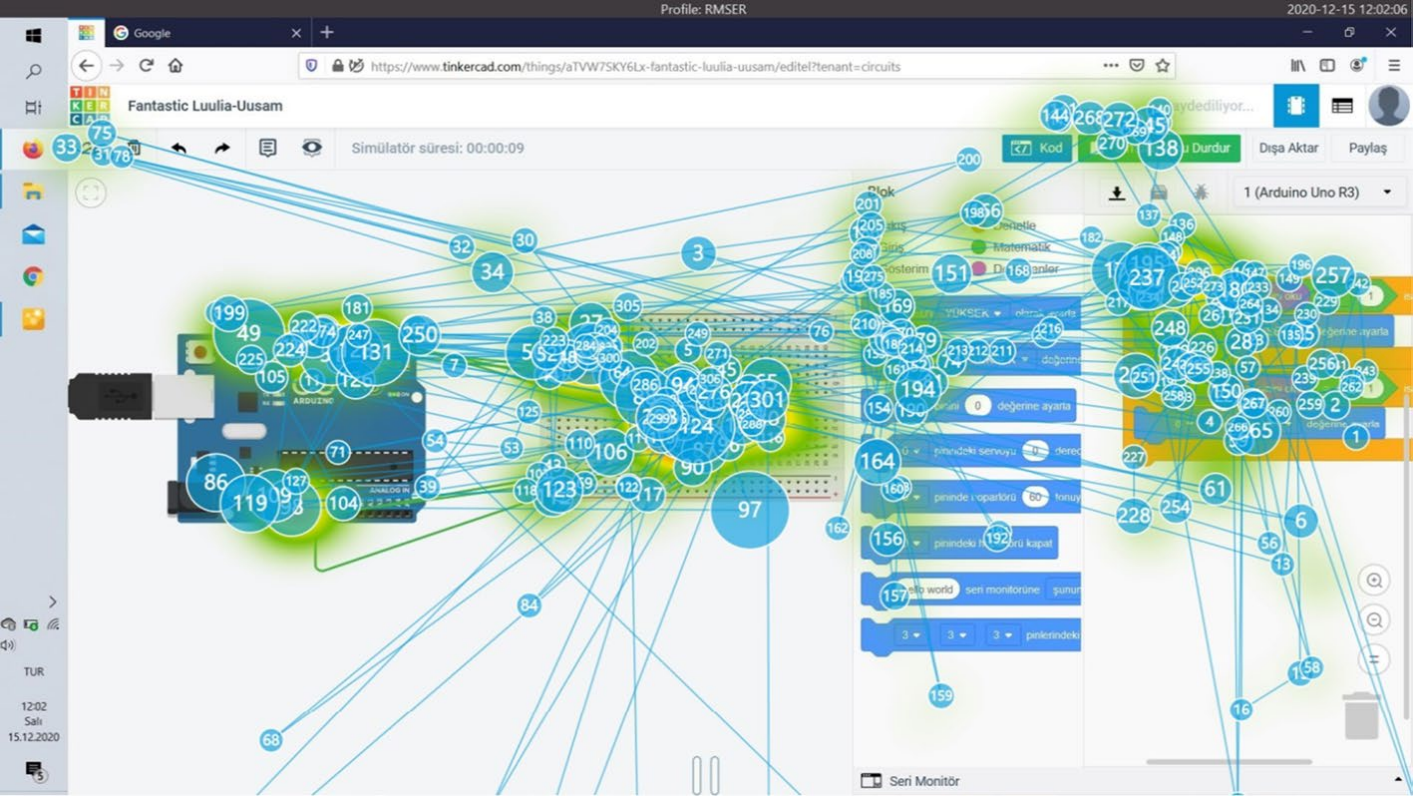
Task 5 heat and splash map of participant 6

When Fig. 8 is examined, it can be seen that participant 6 has a lot of eye splashes during performing the task and constantly scans to find the correct code block on the codes. The excess of eye splashes indicates that the user searches too much on the screen. The task in the "very difficult" category with an average completion time is Task 5, the average completion time of the task is 348 s. The heat and splash map for Participant 6, who completed the task in the shortest time of 196 s, is given in Fig. 9.

**Fig. 10:**
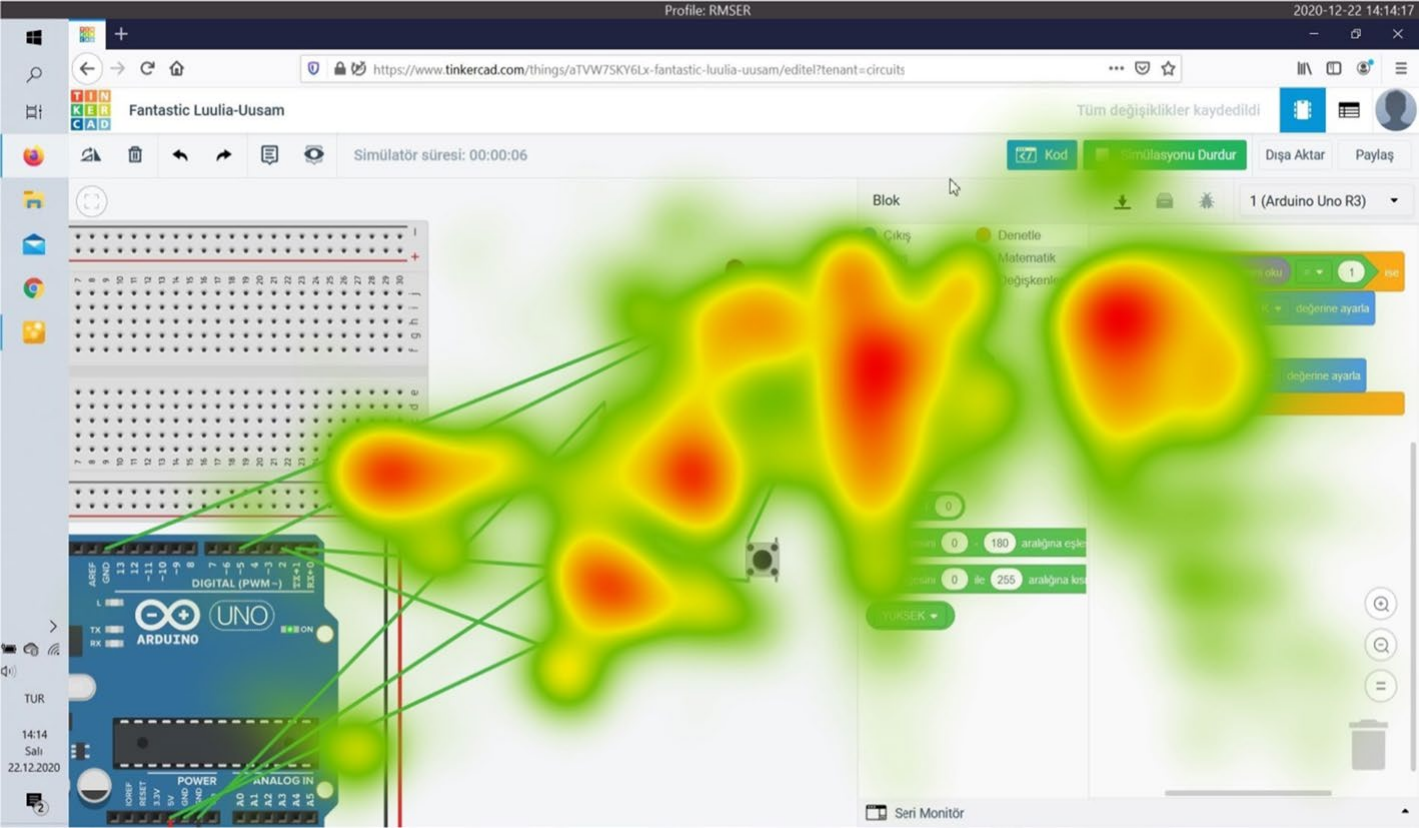
Task 5 heat map of participant 12

**Fig. 11:**
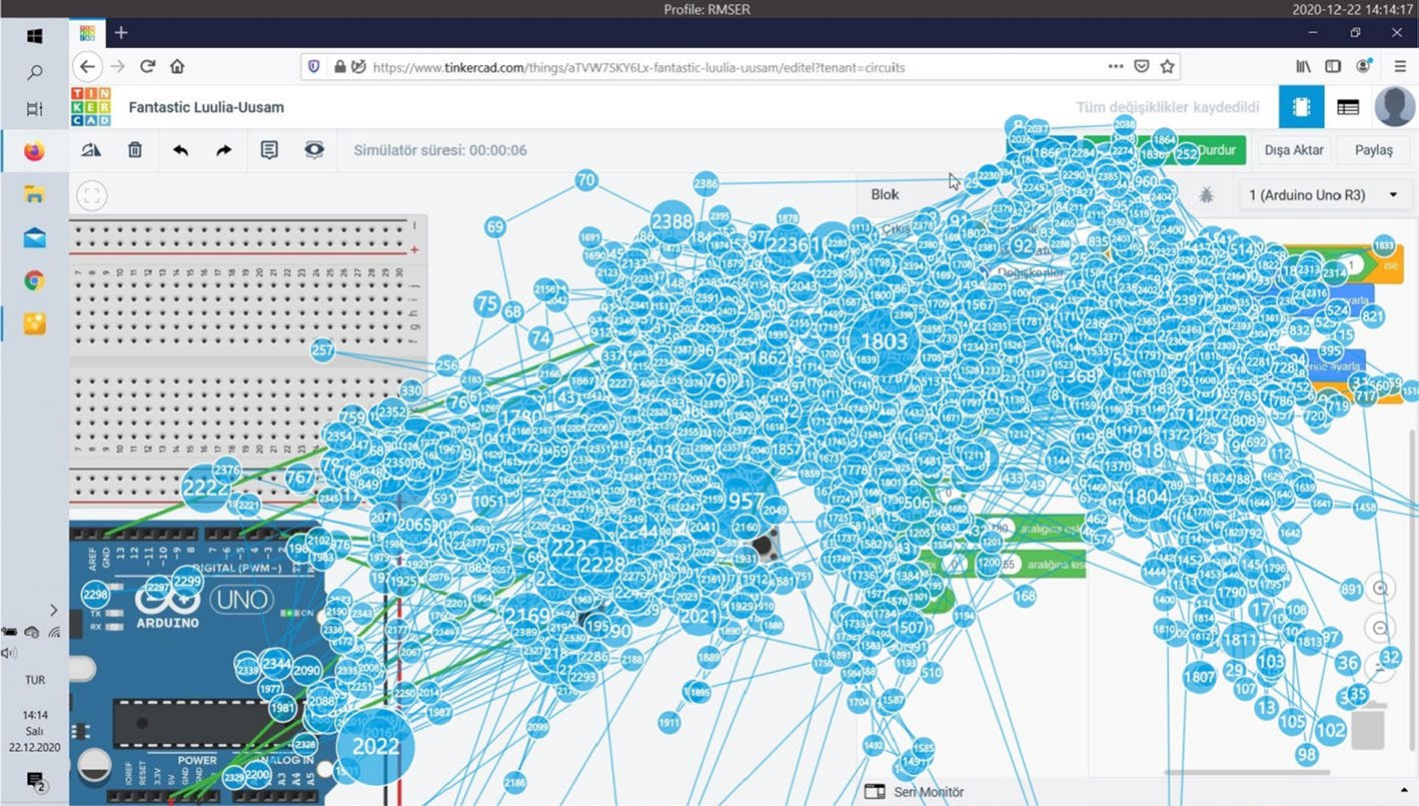
Task 5 splash map of participant 12

When Fig. 9 is examined, it is determined that the participant who successfully completed this task in the longest time is Participant 12. Completing the task in 951 s, the participant did not use a breadboard while making connections and felt the need to check the connections over and over again. The participant spent a lot of time rebuilding the connections after seeing the codes not working. Figure 10 shows the heat map and Fig. 11 shows the splash map of Participant 12.

The splash map shows how much effort the participant has spent, and the heat map shows that most of the connections and code execution are concentrated on the screen (Fig. 11).

**Fig. 12:**
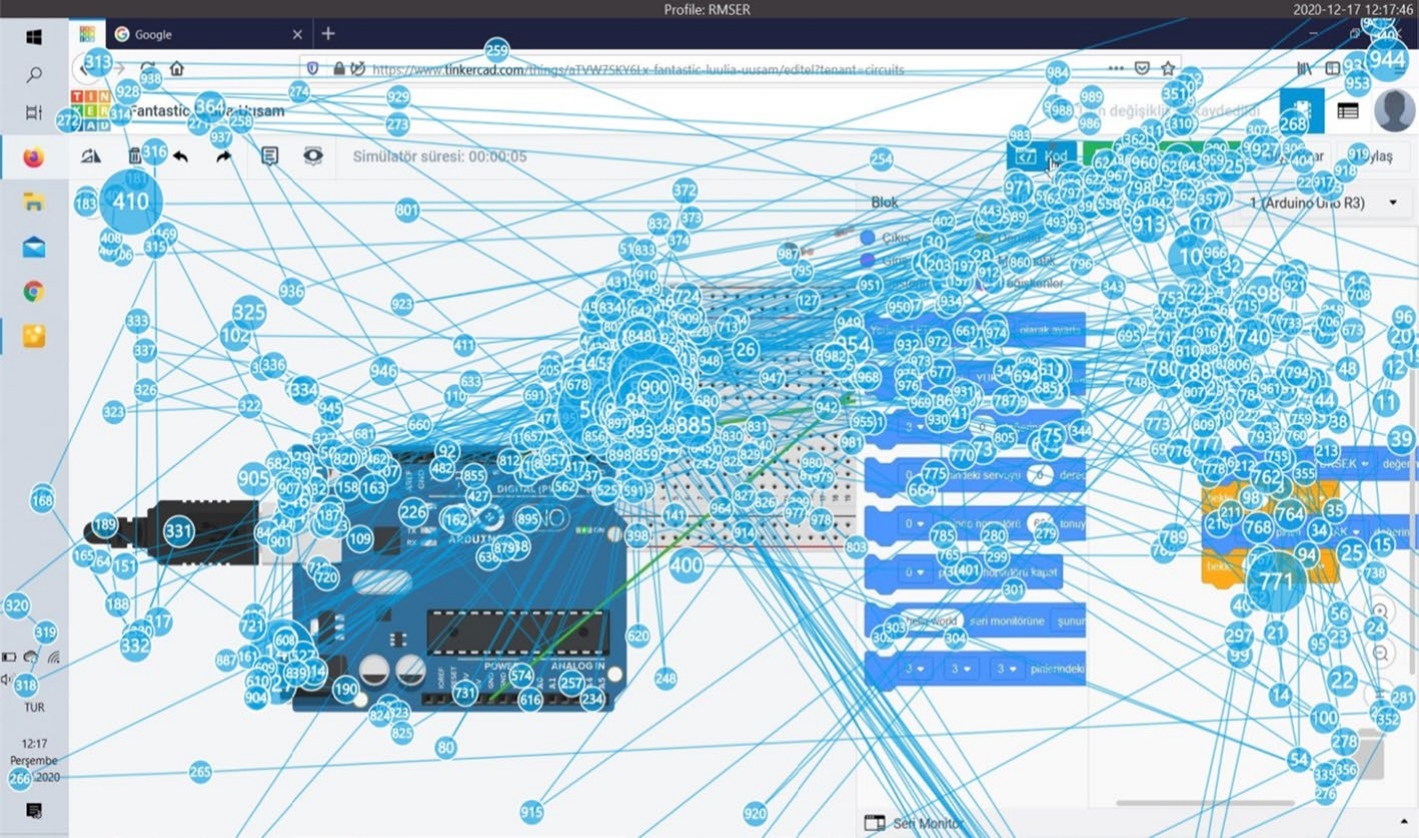
Task 1 splash map for participant 6

Although Task 1 is "very easy", the average completion time is 171 s. It is thought that the task took a long time because the participants first started the application with this task, and the process of getting used to the Tinkercad circuits section was overcome with this task. The participant who completed this task in the longest time was Participant 6 with 575 s. Figure 12 shows the splash map of User 6.

When Fig. 12 is examined, it is seen that the user scans the screen a lot while performing the task and spends a lot of effort while creating the circuit diagram and codes.

**Fig. 13:**
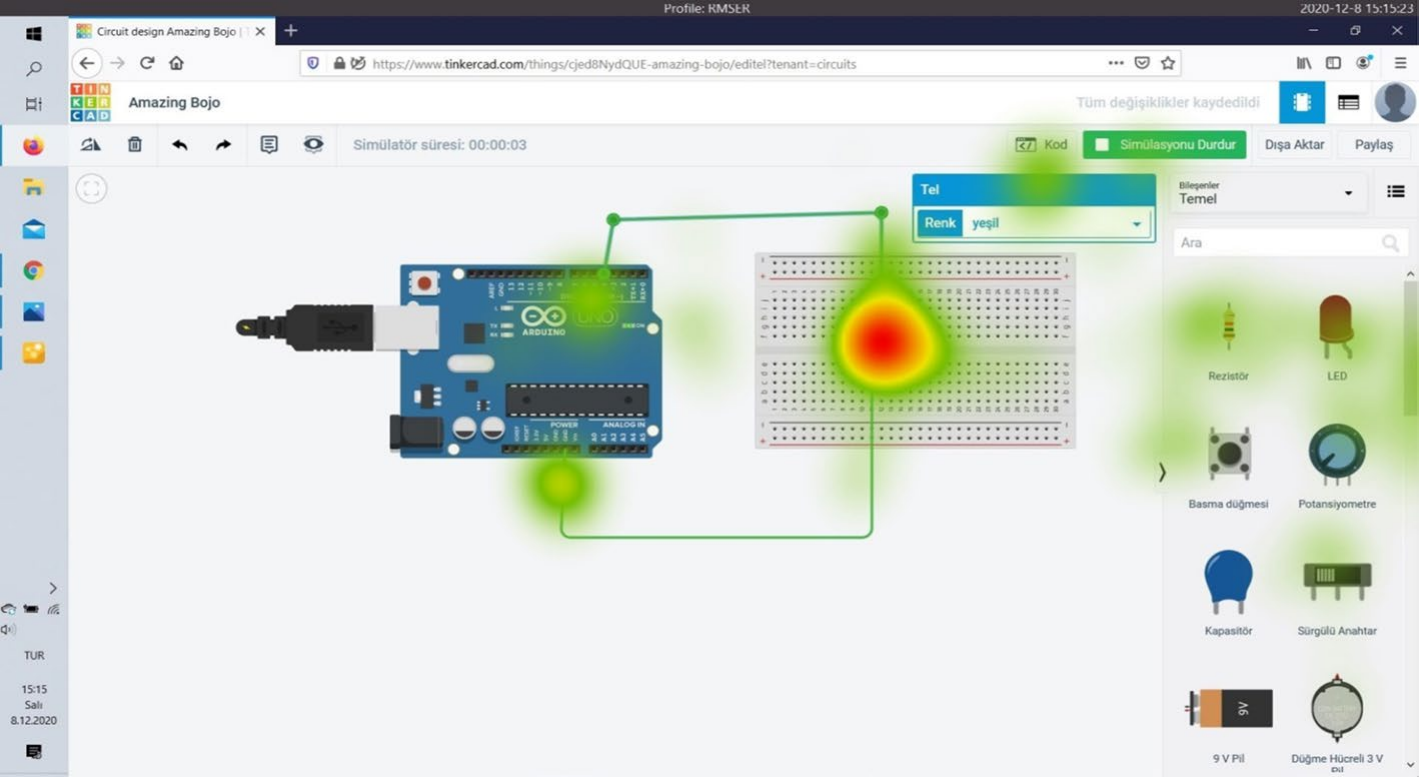
Task 1 heat map of participant 1

**Fig. 14:**
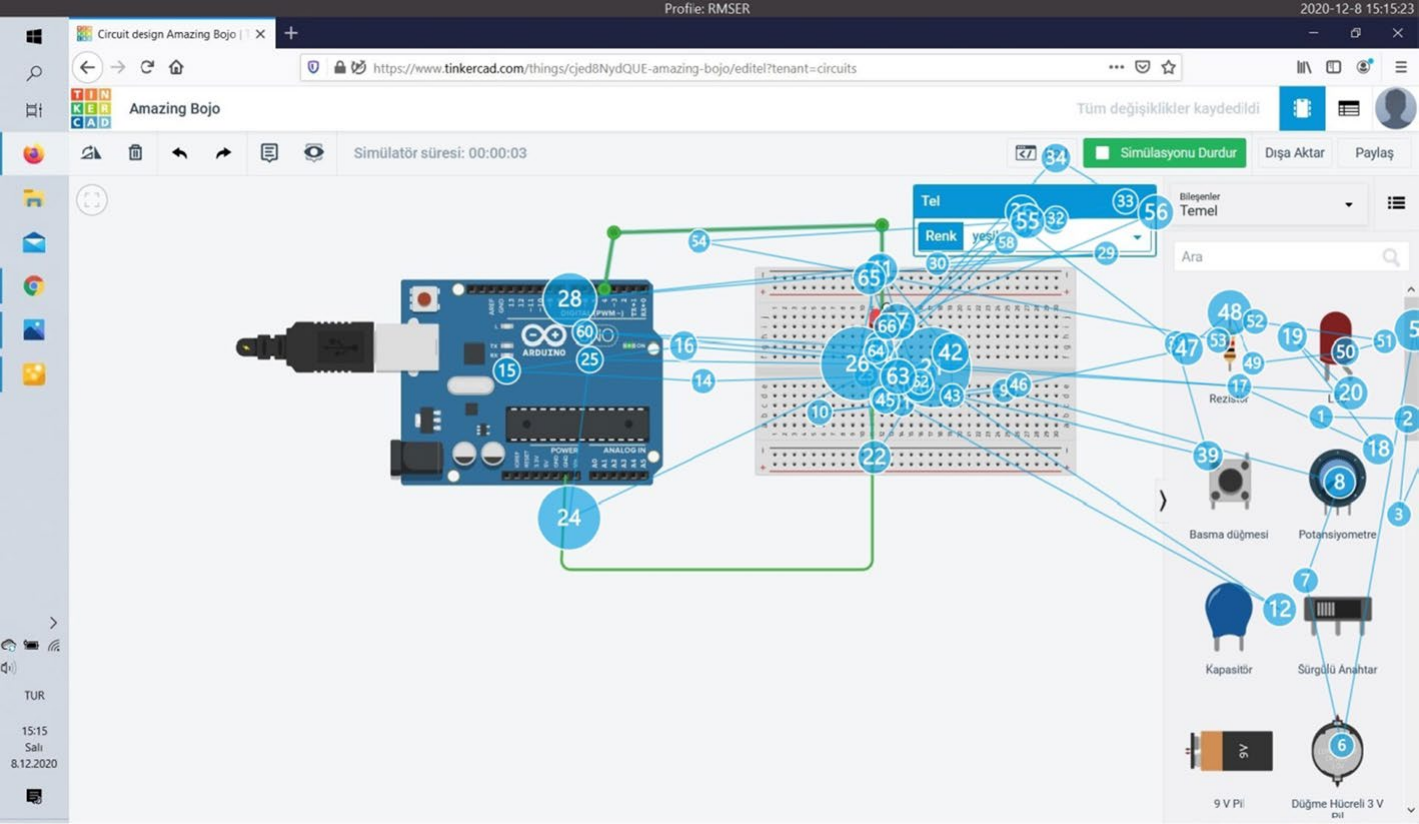
Task 1 splash map of participant 1

The user who completed task 1 in the shortest time, Participant 1 completed the task in 52 s and successfully ran the simulation. The heat map of the user is given in Fig. 13, and the splash map is given in Fig. 14.

All participants who completed the tasks were asked to fill in a survey form after the application. Participants were asked which tasks they did the easiest and which tasks they had difficulties doing. The participants, who stated the most difficult task as Task 5, stated that they had difficulties due to the difficulty of the connections and the inability to remember the codes. The users who specified Task 6 as the easiest task said that although they have never used the Mikro:bit before, it is easy to use, and its coding is simple. Participants stated that they can use Tinkercad in their lessons, recommend them to other teachers and students, and it is suitable for children aged 10 and over.

## Discussion

Online learning environments are getting more and more important day by day and the user base is increasing. In particular, within the scope of the Covid-19 processes, many pieces of training continue remotely, apart from compulsory education, and virtual learning platforms are more needed. In the days of digitalization, many people, including adults, strive to learn to program. Teachers strive to raise their students' awareness of problem-solving, algorithmic skills, coding skills, and robotic skills. Teachers who want to continue their education with Arduino and Micro:bit, which are the most widely used tools in the area of robotics coding, need virtual simulators in distance education. However, research on which tool to use from many simulators will be a waste of time for teachers. In this study, a virtual simulator that teachers can use in the classroom environment during and after distance education was tested in terms of usability.

In the review of the literature, it is seen that usability studies of many online platforms and virtual learning areas were conducted. However, usability studies of the Tinkercad platform or any robotic coding simulation application could not be found. This study is the first study examining the usability of the Tinkercad platform, it contributes to the literature since there is no other study about the usability of virtual circuit simulators and it is the first. Recommending a platform that is easy to use and whose effectiveness and efficiency have been tested reveals the importance of the study. In addition, this study is a recommendation in terms of revealing the deficiencies of the platform by noting the problems encountered by the users during the tests, eliminating the deficiencies, and contributing to the development of the platform.

### Conclusion, and recommendations

As a result of the application tests in which 12 information technologies teachers participated; It was observed that 11 participants who included robotic coding studies with Arduino microcontroller cards in their lessons and activities completed the tasks to a large extent. In addition, 10 participants had no difficulty in performing the tasks, although they had never used the Micro:bit microcontroller card before. It was observed that the participants could easily find the circuit elements they were looking for in the category of Tinkercad circuits and were successful in coding because it was block-based coding. In this case, it was determined that the Tinkercad platform was easy to use by the information technology teachers.

It is known that the factors of effectiveness, efficiency, and satisfaction are important in measuring usability (Cagiltay, 2011). When we examined the level of completion of the tasks for effectiveness, it was found that 9 out of 10 tasks were completed. It was observed that the participants completed their tasks at an average rate of 89%. For this reason, it can be said that the circuits category of the Tinkercad platform is 89% usable by information technology teachers.

Usability studies conducted separately for different user groups such as teachers and students are effective in the development of the application (Emiroglu, 2019). The usability research of the platform can be expanded by conducting usability tests of the Tinkercad circuits section with children over 10 years old. Arduino is flexible in circuit design and programming (Yenikalayci & Harman, 2020). For this reason, Arduino and similar microcontrollers are used in different courses. Similar applications can be made to science or physics teachers who include electrical and circuitry topics in their courses. It is also possible to create 3D designs on the Tinkercad platform. It is preferred not only in information technology courses but also in other fields. 3D learning objects are effective in embodying abstract concepts and perpetuating learning (Tasti et al., 2015). It is thought that the use of content created with Tinkercad in the educational environment will arouse curiosity in students and contribute to the development of creative skills and imagination (Dogan & Uluay, 2020). In this context, the usability test of the 3D design category, which is also used by different branch teachers in future studies, can be performed and the positive and negative aspects of the platform can be revealed.

It is also necessary to express the common difficulties encountered in the use of the Tinkercad platform during user tests. In the circuits section, the circuit elements added to the creation area of the circuits have to be deleted one by one when a new circuit is created, it may be more convenient to clean the circuit area in a shorter time. In addition, after the circuit is created, the circuit elements are closed when writing the code blocks, and the code blocks part can be closed automatically when running the simulation. Blocks are too large when building blocks in the code blocks section, making it difficult to see them in the coding area.

## Data Availability

Data for this study were collected through a survey, observation form and eye tracking device. These data supporting the findings of this study are available upon request from the first and corresponding author.
